# Factors associated with selective fiberoptic bronchoscopy use after left double-lumen tube placement

**DOI:** 10.1186/s12871-026-03855-3

**Published:** 2026-04-22

**Authors:** Onur Küçük, Musa Zengin, Hilal Sazak, Oya Kaybal, Gülay Ülger, Ramazan Baldemir, Mehtap Tunç, Fatma Öztürk Yalçın, Ali Alagöz

**Affiliations:** 1 Department of Anesthesiology and Reanimation, Dokuz Eylül University, School of Medicine, İzmir, Türkiye; 2 Department of Anesthesiology and Reanimation, Ankara Etlik City Hospital, University of Health Sciences, Ankara, Türkiye; 3 Department of Anesthesiology and Reanimation, Ankara Atatürk Sanatoryum Training and Research Hospital, University of Health Sciences, Ankara, Türkiye

**Keywords:** Fiberoptic bronchoscopy, Double-lumen tube, Thoracic anesthesia, Tube malposition, Operator experience

## Abstract

**Background/aim:**

Fiberoptic bronchoscopy (FOB) is considered the gold standard for confirming correct placement of left-sided double-lumen tubes (LDLTs) during thoracic anesthesia. However, routine FOB use is not always feasible in high-volume centers, and selective strategies are frequently adopted. This study aimed to identify factors associated with the need for selective intraoperative FOB following conventional LDLT placement in a high-volume thoracic anesthesia practice where FOB is not routinely used.

**Methods:**

This retrospective observational study was conducted in a high-volume tertiary thoracic surgery center over a 3.5-year period (July 2017 to January 2021). Adult patients undergoing elective thoracic surgery with LDLT placement were reviewed. FOB was not used routinely and was performed only when clinical findings suggested possible tube malposition. Patients were categorized according to intraoperative FOB requirement. Controls were randomly selected in a 1:2 ratio. Demographic, clinical, surgical, and operator-related variables were evaluated. Binary logistic regression was used to identify independent predictors of FOB requirement.

**Results:**

Among 2155 eligible patients, FOB was required in 152 (7.05%); 136 patients with complete data were analyzed. Compared with controls (*n* = 272), patients requiring FOB more frequently had higher body weight, higher body mass index, smaller LDLT size, and procedures performed by less experienced anesthesiologists. In multivariable analysis, anesthesiologist experience remained the only independent predictor of FOB requirement: procedures performed by anesthesiologists with < 1 year (OR 6.77; 95% CI 3.66–12.52; *p* < 0.001) and 1–5 years of experience (OR 2.57; 95% CI 1.43–4.60; *p* = 0.001) were associated with increased FOB use. The most common indications for FOB were pressure and/or volume incompatibility and unexpected desaturation, with malposition most frequently corrected via the tracheal lumen.

**Conclusion:**

In thoracic anesthesia practices where routine FOB is not available, a selective, experience-guided FOB strategy appears appropriate. Anesthesiologist experience is the primary determinant of intraoperative FOB requirement following LDLT placement, whereas patient- and procedure-related factors do not independently predict the need for bronchoscopy. A lower threshold for FOB use should be considered when LDLT placement is performed by less experienced anesthesiologists.

## Introduction

One-lung ventilation (OLV) is an essential component of anesthesia management for a wide range of thoracic surgical procedures, including pneumonectomy, lung resections, and video-assisted thoracic surgery (VATS) [1, 2]. Although bronchial blockers may also be used to achieve lung separation, the left-sided double-lumen tube (LDLT) remains the most commonly preferred and widely accepted technique due to its reliability and versatility in thoracic anesthesia practice [3, 4].

Despite its widespread use, correct placement of an LDLT requires specific technical expertise and remains challenging [5]. Malposition of the LDLT is a well-recognized problem and may result in clinically significant complications such as hypoventilation, hypoxemia, ventilation–perfusion mismatch, insufficient collapse of the nondependent lung, and interruption or prolongation of the surgical procedure due to inadequate lung isolation [6, 7].

Several methods have been described to confirm correct LDLT positioning, including inspection of chest wall movements, auscultation, ventilator parameter assessment, and lung ultrasonography [8–12]. Among these techniques, fiberoptic bronchoscopy (FOB) is widely regarded as the gold standard for confirming and correcting LDLT position [5, 13, 14]. In line with contemporary recommendations from the EACTAIC Thoracic Anesthesia group, bronchoscopic visualization through the tracheal lumen is recommended to verify correct DLT positioning [15]. However, routine FOB use for every patient may not always be feasible in daily clinical practice, particularly in high-volume centers, owing to limitations related to equipment availability, time constraints, cost, and the need for experienced personnel [13, 14].

As a result, many institutions adopt a selective FOB strategy, in which FOB is performed only when clinical signs raise suspicion of tube malposition. Nevertheless, even when conventional clinical methods are initially successful, LDLT malposition rates remain considerable [6, 7]. Common malposition patterns include distal advancement of the bronchial cuff, proximal displacement with cuff herniation above the carina, and inadvertent intubation of the right main bronchus [7, 16]. These malpositions may occur due to excessive bronchial cuff inflation, patient repositioning, intraoperative surgical manipulation, or anatomical variability [17–19].

Although previous studies have primarily focused on comparing confirmation techniques, evaluating alternative devices, or advocating routine FOB use [20–24], limited data are available regarding which patients or clinical circumstances are more likely to require FOB when a selective strategy is employed. In particular, the relative contribution of patient-related factors and operator experience to the need for clinically indicated FOB following LDLT placement has not been sufficiently clarified [25–28].

Therefore, the present study aimed to identify factors associated with the need for selective FOB following LDLT placement in a high-volume thoracic anesthesia practice where FOB is not routinely used. We assessed whether the need for FOB following LDLT placement would be influenced by a combination of patient-related and operator-related factors, rather than occurring randomly, and that specific high-risk profiles could be identified.

## Materials and methods

### Study design and ethical approval

This retrospective observational study was conducted in a high-volume tertiary thoracic surgery center after approval by the local Ethics Committee (Approval No: 2012-KAEK-15/2314, Date: 25/05/2021). Adult patients who underwent elective thoracic surgery between July 2017 and January 2021 were included. Due to the retrospective nature of the study, the requirement for informed consent was waived by the Ethics Committee.

### Study setting

This study was conducted in a high-volume tertiary referral center dedicated exclusively to thoracic surgery with multiple operating rooms performing elective thoracic procedures daily. During the study period, routine fiberoptic bronchoscopy (FOB) confirmation after left double-lumen tube (LDLT) placement was not mandated by institutional protocol. Although FOB is widely recommended as the gold standard for confirming LDLT position, its use in our center was selective during the data collection period and based on clinical indicators suggestive of malposition.

Due to the high surgical turnover and limited simultaneous availability of bronchoscopic equipment, LDLT placement was initially performed using conventional clinical assessment methods. In cases where clinical findings indicated possible malposition, FOB was promptly performed for confirmation and correction. This approach reflects a real-world practice model in high-volume centers where selective bronchoscopic verification is used instead of routine bronchoscopic verification, and in centers where access to FOB is challenging.

### Patient selection

Anesthesia records and intraoperative documentation were retrospectively reviewed for patients aged 18–80 years with American Society of Anesthesiologists (ASA) physical status I–III who underwent elective thoracic surgery requiring OLV using a LDLT. Patients were excluded if a single-lumen tube or bronchial blocker or right DLT was used, if they were younger than 18 years, if emergency surgery was performed, or if anesthesia records or intraoperative data were incomplete, including missing information regarding FOB use.

### Study groups

Patients were categorized into two groups according to the intraoperative requirement for FOB:

- Group 1: patients who required intraoperative FOB due to clinical suspicion of LDLT malposition.
- Group 2: patients who did not require FOB.

For comparative analysis, patients in Group 2 were randomly selected from the eligible cohort using a computer-generated randomization method, with a 1:2 ratio relative to Group 1. This ratio was chosen because statistical power increases substantially when the control-to-case ratio is increased from 1:1 to 1:2, while marginal gain beyond this threshold is minimal [29]. With 136 complete FOB cases among 2155 eligible patients, a 1:2 random sample provided near-equivalent power to full cohort analysis.

### Data collection

Demographic variables including age, sex, height, weight, and body mass index (BMI) were recorded. BMI was categorized as < 25 kg/m², 25–29.9 kg/m², and ≥ 30 kg/m². Clinical and surgical data included the primary diagnosis, type and side of thoracic surgery, and history of previous thoracic surgery. Airway management–related variables comprised LDLT size and the anesthesiologist’s experience, categorized as < 1 year, 1–5 years, and ≥ 5 years. All procedures were performed by fully trained anesthesiologists who had completed their residency in anesthesiology and reanimation. This classification therefore referred to the duration of post-residency clinical practice, not to thoracic anesthesia exposure or the number of LDLT placements performed. Procedural case volume was not recorded in institutional anesthesia records and could not be retrieved retrospectively. In patients who required FOB, additional data were collected regarding the indication for FOB, timing of FOB application, type of LDLT malposition, and the corrective maneuvers performed.

### LDLT placement and confirmation protocol

After standard monitoring and preoxygenation, anesthesia induction and mask ventilation were performed according to institutional practice. LDLT placement was achieved using the conventional technique. Following direct laryngoscopy, the bronchial tip of the LDLT was advanced beyond the vocal cords, the stylet was removed, and the tube was rotated 90° counterclockwise before being advanced until mild resistance was encountered. Correct tracheal intubation was confirmed using continuous capnography.

Initial confirmation of LDLT position was performed using clinical methods, including chest wall inspection, auscultation, and assessment of ventilatory parameters. FOB was not used routinely. When malposition was suspected based on clinical findings such as inadequate ventilation, unexpected desaturation, abnormal airway pressures, or insufficient lung collapse, FOB was performed to evaluate tube position and guide corrective maneuvers. Clinical indicators prompting FOB were predefined based on routine institutional clinical practice and included unexplained increases in airway pressure, unexpected reductions in delivered tidal volume during OLV, inadequate lung collapse observed by the surgeon, persistent oxygen desaturation not attributable to other causes, or discordance between expected and measured ventilatory parameters after lateral repositioning.

Bronchoscopic assessment of LDLT position was based on two mandatory endpoints as described by Bussières and Slinger [5]: an unobstructed view of the left upper and lower lobe bronchial orifices through the bronchial lumen, and visualization of the bronchial cuff positioned below the tracheal carina through the tracheal lumen. Malposition was defined as any tube position requiring corrective movement to achieve these endpoints. Three bronchoscopic malposition patterns were recorded: distal displacement, defined as advancement of the bronchial tip beyond the secondary carina with loss of left upper lobe orifice visualization; proximal displacement, defined as herniation of the bronchial cuff above the tracheal carina; and advancement into the right main bronchus, defined as misdirection of the bronchial tip into the right mainstem bronchus with loss of left-sided isolation [5, 22, 25]. All FOB procedures were carried out by anesthesiologists experienced in bronchoscopy.

### Outcomes

The primary outcome was the requirement for clinically indicated intraoperative FOB following LDLT placement. Secondary outcomes included the indications for FOB, bronchoscopic findings and types of malposition identified among patients who underwent FOB, timing of FOB application, and the corrective methods used to restore appropriate tube positioning.

### Statistical analysis

Statistical analyses were performed using SPSS for Windows, version 22.0 (IBM Corp., Armonk, NY, USA). The normality of continuous variables was assessed using the Shapiro-Wilk test, and homogeneity of variances was evaluated with the Levene test. Continuous variables were presented as mean ± standard deviation (SD) for normally distributed data or median (interquartile range; IQR) for non-normally distributed data, while categorical variables were expressed as number (n) and percentage (%).

Between-group comparisons were conducted using the Student’s t-test or Mann–Whitney U test for continuous variables, as appropriate. Categorical variables were compared using the Pearson chi-square test or Fisher’s exact test. To identify independent predictors of intraoperative FOB requirement, binary logistic regression analysis was performed. Variables considered clinically relevant or statistically significant in univariable analysis were entered into the multivariable model. Results were reported as odds ratios (ORs) with 95% confidence intervals (CIs). Model discrimination was evaluated using the area under the receiver operating characteristic curve (AUC), with a default probability cutoff of 0.5.

A p-value < 0.05 was considered statistically significant for all analyses.

## Results

### Study population

**Fig. 1:**
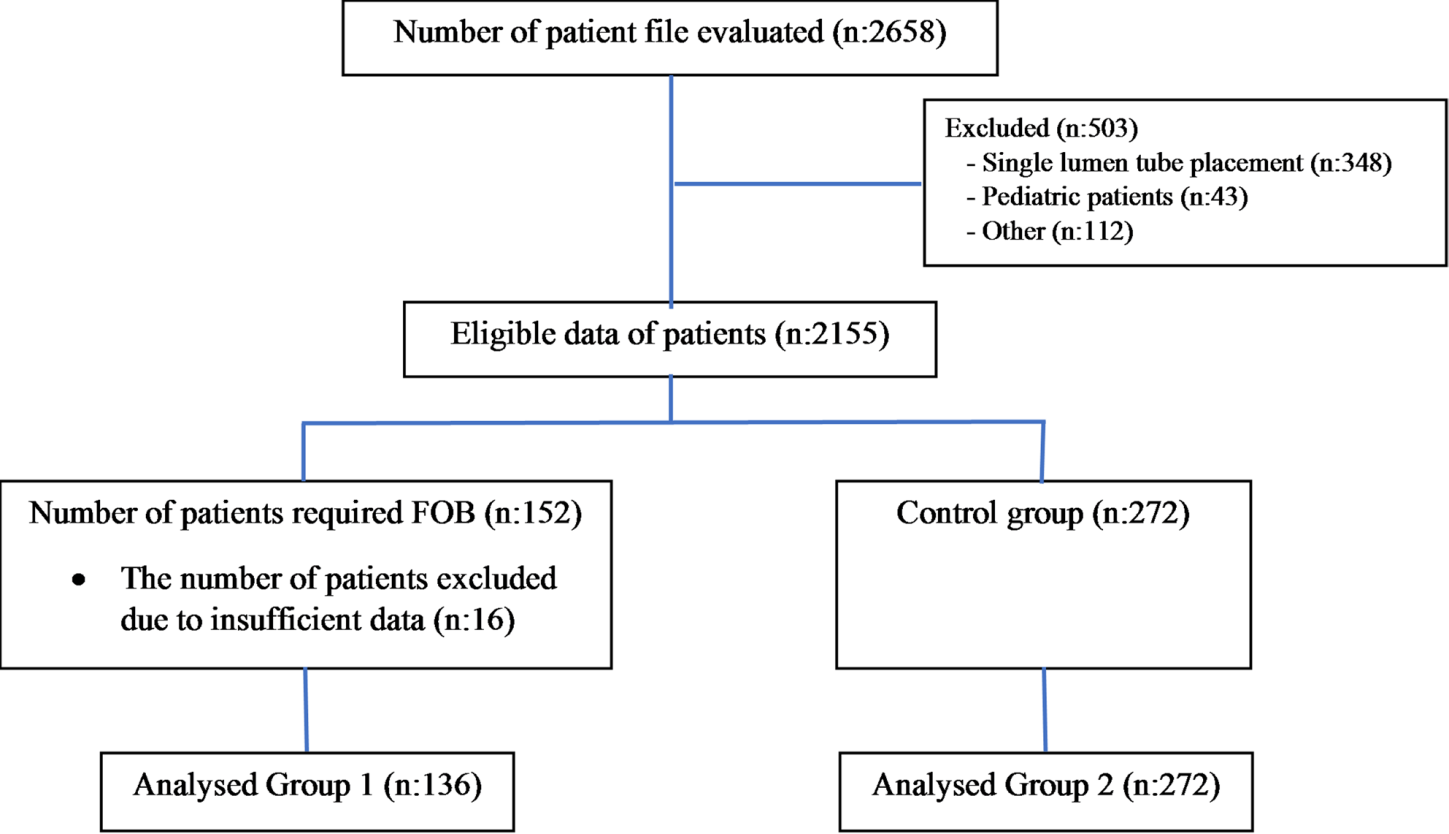
Flow charts of the patients. FOB: Fiberoptic bronchoscopy

A total of 2658 patient files were screened. After exclusions (*n* = 503), 2155 adult patients who underwent elective thoracic surgery with LDLT placement were eligible. FOB was required in 152 (7.05%) patients; 16 were excluded due to insufficient data, leaving 136 patients (6.3%) for analysis (Group 1). A 1:2 random sample of patients who did not require FOB (*n* = 272) was selected as the control group (Group 2) (Fig. 1).

### Baseline characteristics and operator experience

Female sex was more frequent in Group 1 (43.4% vs. 29.4%, *p* = 0.005). Median body weight was higher in Group 1 (78 vs. 74 kg, *p* = 0.033). Median BMI was also higher (28.8 vs. 26.4 kg/m², *p* = 0.009), and obesity (BMI ≥ 30 kg/m²) was more common (38.2% vs. 26.8%, *p* = 0.043) (Table 1).

**Table 1:** Demographic characteristics, physician experiences, and diagnostic features of patients

Variables		Group 1 (*n*:136)	Group 2 (*n*:272)	*p*
Age, year		59 (16.3)	59 (17)	0.950^Φ^
Gender	Female	59 (43.4%)	80 (29.4%)	0.005*
	Male	77 (56.6%)	192 (70.6%)	
Weight, kg		78 (22)	74 (20)	**0.033** ^Φ^
Height, cm		166 (13)	168 (12)	0.081^Φ^
	< 160	30 (22.1%)	40 (14.7%)	0.162*
	160–170	63 (46.3%)	132 (48.5%)	
	> 170	43 (31.6%)	100 (36.8%)	
BMI, kg/m^2^		28.8 (7.87)	26.4 (7.24)	**0.009** ^Φ^
	< 25 kg/m^2^	42 (30.9%)^a^	111 (40.8%)^b^	0.043*
	≥ 25–30 kg/m^2^	42 (30.9%)^a^	88 (32.4%)^a^	
	≥ 30 kg/m^2^	52 (38.2%)^a^	73 (26.8%)^b^	
Anesthesiologist experience	≥ 5 years	21 (15.4%)^a^	113 (41.5%)^b^	< 0.001*
	1–5 years	53 (39.0%)^a^	105 (38.6%)^a^	
	< 1 year	62 (45.6%)^a^	54 (19.9%)^b^	
History of previous Thoracic Surgery	Yes	12 (8.8%)	28 (10.3%)	0.638*
	No	124 (91.2%)	244 (89.7%)	
Diagnosis	Malignancy	94 (69.1%)	177 (65.1%)	0.280*
	Bronchiectasis	9 (6.6%)	13 (4.8%)	
	Hydatid cyst	8 (5.9%)	10 (3.7%)	
	Pleural pathologies	11 (8.1%)	38 (14%)	
	Pneumothorax	5 (3.7%)	19 (7%)	
	Other	9 (6.1%)	15 (5.4%)	

Continuous variables are expressed as median (interquartile range)^Φ﻿^, while categorical variables are expressed as either frequency (percentage)*. Continuous variables were compared with the Student t-test or Mann-Whitney u test, and categorical variables were compared using Pearson’s Chi-Square test or Fisher exact test. Statistically significant *p*-values are in bold. a vs. b: Significant difference between the groups. a vs. a: No significant difference between the groups

*BMI* Body mass index

The distribution of anesthesiologist experience differed markedly between groups (*p* < 0.001): cases performed by anesthesiologists with ≥ 5 years of experience were less common in Group 1 (15.4% vs. 41.5%), whereas cases performed by anesthesiologists with < 1 year of experience were more common (45.6% vs. 19.9%) (Table 1).

### Surgical characteristics and DLT size

There was no significant difference in operated side (*p* = 0.153) or the distribution of VATS versus thoracotomy (*p* = 0.397) (Table 2).

**Table 2:** Surgical characteristics of patients and the size of the double-lumen tube

Variables	Group 1 (*n*:136)	Group 2 (*n*:272)	*p*
	*n* (%)	*n* (%)	
Site of Surgery			
Left	48 (35.3%)	116 (42.6%)	0.153*
Right	88 (64.7%)	156 (57.4%)	
Type of Surgery			
VATS	55 (40.4%)	122 (44.9%)	0.397*
Thoracotomy	81 (59.6%)	150 (55.1%)	
Surgical Procedure			
Wedge resection	38 (27.9%)	86 (31.6%)	0.405*
Segmentectomy	62 (45.6%)	105 (38.6%)	
Lobectomy	12 (8.8%)	29 (10.7%)	
Pneumonectomy	11 (8.2%)	27 (9.9%)	
Decortication	4 (2.9%)	15 (5.5%)	
Cystotomy and capitonnage	9 (6.6%)	10 (3.7%)	
DLT Size			
35	49 (36%) ^a^	56 (20.6%) ^b^	0.008*
37	20 (14.7%) ^a^	42 (15.4%) ^a^	
39	41 (30.1%) ^a^	108 (39.7%) ^a^	
41	26 (19.1%) ^a^	66 (24.3%) ^a^	

Categorical variables were expressed as either frequency (percentage)*. Categorical variables were compared using Pearson’s Chi-square test or Fisher exact test. Statistically significant *p*-values are in bold. a vs. b: Significant difference between the groups. a vs. a: No significant difference between the groups

*VATS* Video-assisted thoracoscopic surgery, *DLT* Double Lumen Tube

DLT size differed between groups: 35 Fr tubes were used more frequently in Group 1 (36.0% vs. 20.6%, *p* = 0.008) (Table 2).

### Indications for FOB, findings, timing, and corrective actions (Group 1)

Among patients requiring FOB (Group 1), the most common indication was pressure and/or volume incompatibility in the dependent lung (50.7%), followed by unexpected desaturation (25.0%), initial suspicion of malposition (20.6%), and insufficient collapse of the nondependent lung (23.5%) (multiple indications were possible). FOB findings demonstrated malposition in most cases: the DLT was distal to the main bronchus in 29.4%, proximal in 28.7%, and had advanced into the right main bronchus in 27.2%; no malposition was observed in 14.7%. FOB was performed in the supine position in 38.9% and after lateral positioning / intraoperatively in 61.1%. Corrective actions were most commonly performed via the tracheal lumen (57.4%), followed by the bronchial lumen (27.2%); 15.4% required no correction (Table 3).

**Table 3:** FOB indication, FOB findings, FOB timing, and malposition correction method

Variables	Group 1 (*n*:136; 6.3%)
	*n* (%)
^a^FOB indication	
^b^Initial Suspicion of Malposition	28 (20.6%)
Pressure and/or Volume Incompatibility in Dependent Lungs	69 (50.7%)
Unexpected Low Saturation	34 (25.0%)
Insufficient Collapse of Nondependent Lung	32 (23.5%)
FOB Findings	
DLT distal to the main bronchus	40 (29.4%)
DLT Proximal to the main bronchus	39 (28.7%)
DLT has involuntarily advanced to the right main bronchus	37 (27.2%)
Malposition not observed	20 (14.7%)
FOB Timing	
Supine	53 (38.9%)
LDP and during surgery	83 (61.1%)
Malposition correction method	
Fixed from the bronchial lumen	37 (27.20%)
Corrected from the tracheal lumen	78 (57.4%)
No correction needed	21 (15.4%)

Categorical variables were expressed as either frequency (percentage)

*FOB *Fiberoptic bronchoscopy,* LDP *Lateral decubitus position,* DLT* Double lumen tube

^a^In some patients, the need for FOB has developed due to more than one reason.

^b^Suspicion of malposition in the clinically conventional evaluation of the DLT position in the supine position initially

### Predictors of FOB requirement: logistic regression

In univariate analysis, anesthesiologist experience was strongly associated with FOB requirement. Compared with procedures performed by anesthesiologists with ≥ 5 years of experience, FOB requirement increased in cases performed by anesthesiologists with < 1 year of experience (OR 6.17; 95% CI 3.41–11.16; *p* < 0.001) and with 1–5 years of experience (OR 2.71; 95% CI 1.53–4.80; *p* < 0.001) (Table 4).

**Table 4:** Univariate and multivariate logistic regression modeling for FOB requirements

Prediction variable	Univariate			Multivariate		
	OR	%95 CI	*p*-value	OR	%95 CI	*p*-value
Anesthesiologist experience, year						
< 1	6.17	3.41–11.16	**< 0.001**	6.77	3.66–12.52	**< 0.001**
1–5	2.71	1.53–4.8	**< 0.001**	2.57	1.43–4.6	**0.001**
DLT size, ≤ 35 Fr	2.17	1.37–3.43	**< 0.001**	1.67	0.73–3.79	0.221
Gender, Female	1.83	1.19–2.82	**0.005**	1.48	0.68–3.24	0.319
BMI, ≥ 30 kg/m^2^	1.68	1.08–2.61	**0.019**	1.20	0.74–1.95	0.445

Statistically significant p-values are in bold.* BMI* Body mass index, *CI* 95% confidence interval, *DLT* Double Lumen Tube, *OR* Odds ratio

**Fig. 2:**
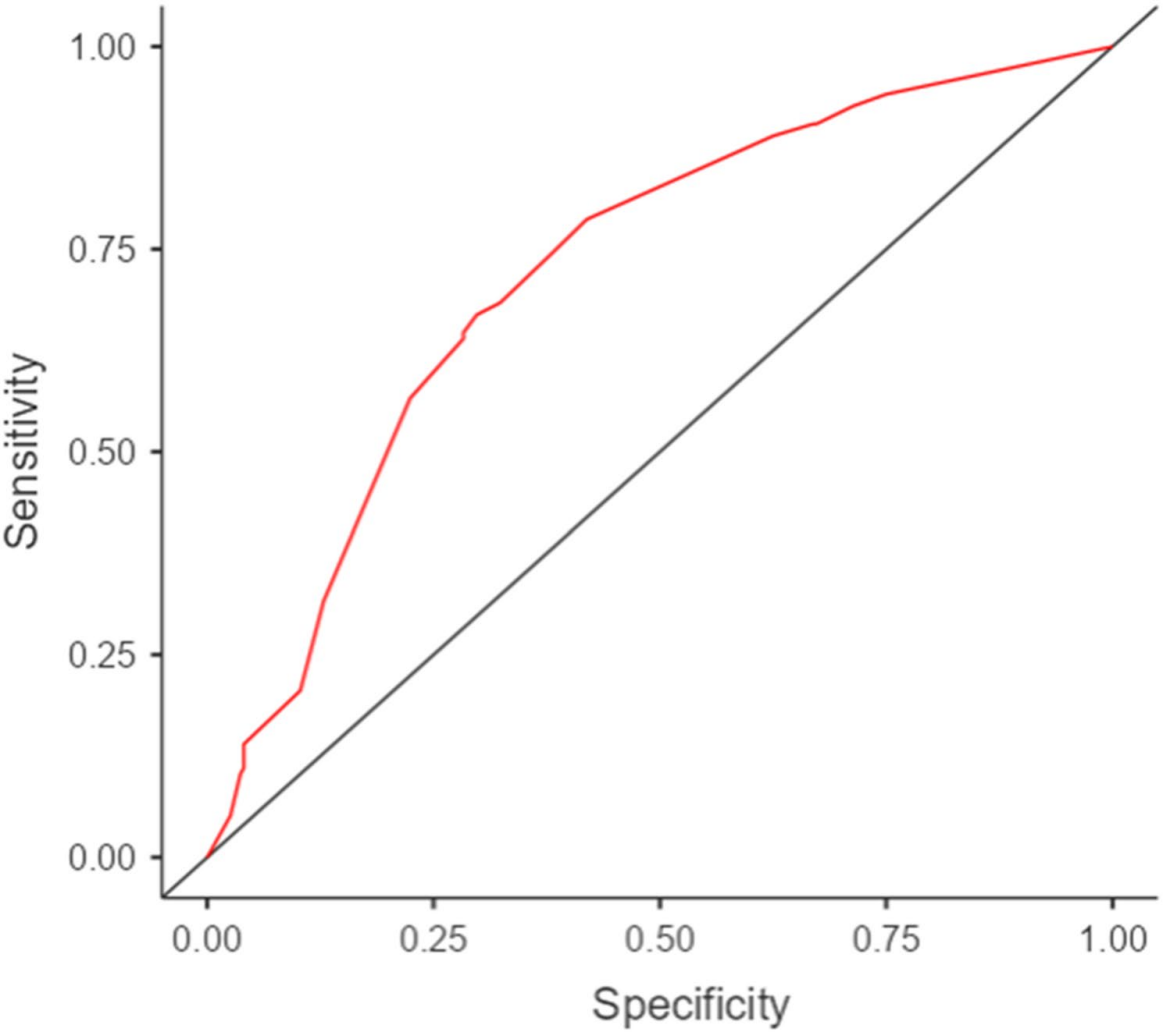
Area under the receiver operating characteristic curve of the model predicting FOB demand. FOB: Fiberoptic bronchoscopy

In the multivariate model (including anesthesiologist experience, sex, BMI category, and DLT size), anesthesiologist experience remained the only independent predictor: <1 year (OR 6.77; 95% CI 3.66–12.52; *p* < 0.001) and 1–5 years (OR 2.57; 95% CI 1.43–4.60; *p* = 0.001) were associated with increased FOB requirement. DLT size ≤ 35 Fr, female sex, and BMI were not independently associated (Table 4). Model performance showed AUC 0.72, with 31.6% sensitivity and 87.1% specificity at the default cut-off of 0.5; anesthesiologist experience contributed most to the model (χ²=42.44) (Table 4, Fig. 2).

### Additional experience-based effect estimates

When experience was dichotomized, FOB requirement increased 3.3-fold for anesthesiologists with < 1 year of experience (OR 3.3; 95% CI 2.15–5.3; *p* < 0.001) and 3.8-fold for anesthesiologists with < 5 years of experience (OR 3.8; 95% CI 2.3–6.57; *p* < 0.001) (Table 5).

**Table 5:** Odds ratios for FOB demand in DLT placement according to anesthesiologist experience year; dichotomized experience thresholds

Anesthesiologist experience	OR	95% CI	*p*-value
< 1 year	3.3	2.15–5.3	**< 0.001**
< 5 year	3.8	2.3–6.57	**< 0.001**

Statistically significant p-values are in bold.* CI* 95% confidence interval, *OR* Odds ratio

## Discussion

In centers with high thoracic case turnover, FOB cannot always be applied to every LDLT placement. In this cohort, FOB was performed only when clinical findings raised suspicion of tube malposition. The primary outcome was therefore the need for clinically indicated bronchoscopy, not bronchoscopically confirmed LDLT malposition. The model identifies predictors of clinically triggered FOB rather than predictors of malposition itself. Within this framework, the need for FOB clustered around operator experience: once patient and procedural variables were modeled together, experience was the only independent determinant. This supports an experience-guided selective FOB approach rather than universal bronchoscopy.

LDLT placement remains the most commonly used and reliable method for lung isolation and separation during thoracic surgery, owing to its effectiveness and versatility in facilitating OLV [1, 2, 5]. However, malposition of LDLTs continues to represent a clinically relevant problem, as it may lead to hypoventilation, hypoxemia, ventilation–perfusion mismatch, and interruption of the surgical workflow [5–7]. Although FOB is widely accepted as the gold standard for confirming correct LDLT positioning [14, 30], its routine use is not always feasible, particularly in high-volume centers where equipment availability, time constraints, and the need for experienced personnel may limit universal application [13, 14]. This reality underscores the clinical importance of identifying patients and situations in which FOB is most likely to be required.

In our cohort, several variables, including female sex, higher body weight and BMI, smaller LDLT size, and lower operator experience were more frequently observed among patients requiring FOB in univariable analyses. These findings are broadly consistent with prior reports and reviews emphasizing that anthropometric characteristics and tube size selection can influence LDLT placement and stability [28, 31, 32]. In clinical practice, sex and height are the primary anthropometric parameters guiding DLT size selection, with 37 Fr predominating in women and 39 Fr in men across a multicentre cohort of 909 patients [33]. No published guideline defines an evidence-based standard for individual DLT size selection, and clinical experience remains the dominant driver of this decision [26, 33]. Zani et al. concluded that experience alone is insufficient without objective preoperative airway measurement, and recommended bronchoscopic verification to confirm tube fit and correct misplacements [33]. The Italian intersociety PACTS consensus further endorses specific airway management planning in patients with obesity, a group that was more frequently represented among FOB cases in our cohort [34]. Taken together, these data suggest that the association between smaller DLT size and FOB requirement observed in univariable analysis reflects an experience-mediated sizing decision rather than an intrinsic tube-size risk. Nevertheless, when these variables were evaluated together in a multivariable model, only anesthesiologist experience remained independently associated with FOB requirement, highlighting the dominant role of operator-related factors in the success of conventional LDLT placement. The model demonstrated moderate discrimination (AUC 0.72), with high specificity (87.1%) but low sensitivity (31.6%) at the default cutoff of 0.5. For comparison, lung ultrasound-based models for DLT confirmation have reported AUC values of 0.86, while clinical assessment alone yields an AUC of approximately 0.52 [11]. Our model therefore sits between these benchmarks. At the default cutoff, the model correctly identifies the large majority of patients who will not require FOB, but misses a substantial proportion of those who will. This performance profile limits its use as an individual-level screening tool. The model may be more useful for identifying high-risk situations at the population level, particularly procedures performed by less experienced anesthesiologists, where a lower threshold for FOB use is warranted regardless of other clinical findings.

Experience in thoracic anesthesia has long been recognized as a key determinant of successful lung isolation. Training-focused studies and observational reports have emphasized a learning curve for accurate LDLT positioning, with improved performance as experience increases [4, 35]. In our study, the likelihood of FOB requirement increased substantially when LDLT placement was performed by anesthesiologists with less than five years of experience, and particularly within the first year of practice. These findings suggest that technical proficiency, familiarity with airway anatomy, and anticipation of malposition scenarios improve with experience, thereby reducing the need for rescue bronchoscopy. Importantly, this supports a pragmatic approach in which the threshold for FOB use should be lower when LDLT placement is performed by less experienced anesthesiologists, even in the absence of overt clinical signs [4, 35].

The timing and clinical triggers for FOB application observed in our study provide further insight into mechanisms of LDLT malposition. The need for FOB was most frequently identified after repositioning the patient from the supine to the LDP or during the intraoperative period. Positioning changes and intraoperative factors are known contributors to tube displacement; lateral positioning has also been associated with bronchial cuff pressure changes and clinically relevant migration [16–18]. Accordingly, heightened vigilance is warranted during these critical phases.

Unexpected increases in airway pressure and decreases in tidal volume were prominent clinical indicators prompting FOB in our cohort. These findings emphasize the importance of continuous ventilatory monitoring during OLV. Sudden changes in airway mechanics should raise immediate suspicion for LDLT malposition and prompt timely bronchoscopic evaluation, consistent with evidence that tube location and malposition correlate with impaired oxygenation and intraoperative instability during OLV [5, 6].

Regarding corrective techniques, malposition was most commonly confirmed and corrected by introducing the bronchoscope through the tracheal lumen of the LDLT. This approach offers a wider field of view and facilitates identification of carinal anatomy, allowing efficient repositioning of the bronchial cuff; subsequent inspection through the bronchial lumen can be used to ensure optimal alignment and to exclude lobar obstruction that may be difficult to detect clinically [5, 13]. This stepwise approach is consistent with established recommendations and proved effective in the majority of our cases.

Current guidelines and expert consensus recommend FOB as the gold standard for confirming LDLT position, both after intubation and after lateral repositioning [5, 26, 36]. Auscultation and clinical assessment alone produce unacceptably high rates of undetected malposition and cannot be considered adequate substitutes [5, 30]. The findings of the present study do not challenge this standard. They describe the determinants of clinically indicated FOB use within a real-world selective strategy, not an argument for abandoning routine bronchoscopic confirmation. The literature acknowledges that selective FOB strategies are adopted in high-volume centers where equipment availability, time constraints, and personnel limitations preclude universal application [13, 15, 37]. Within this pragmatic framework, our findings demonstrate that operator experience is the dominant determinant of FOB requirement. A lower threshold for bronchoscopic confirmation is warranted when LDLT placement is performed by less experienced anesthesiologists, and this recommendation is fully consistent with current guideline-based practice. In this context, our findings extend the existing evidence by demonstrating that, within a selective strategy, anesthesiologist experience remained the dominant determinant of intraoperative FOB requirement in multivariable modeling.

### Limitations

This study has several limitations. Its retrospective, single-center design precludes causal inference and limits generalizability, as institutional practices regarding FOB trigger criteria, tube size selection, and operator supervision may differ across centers. Variable selection was restricted to routinely recorded data, which precluded systematic collection of preoperative airway assessment parameters. Difficult airway predictors including Mallampati score, thyromental distance, mouth opening, and tracheobronchial dimensions were not available for analysis. Prospective studies have demonstrated that anatomical variables such as tracheal and bronchial diameter independently predict malposition risk and DLT placement difficulty [7, 32], and that previous thoracic surgery is an independent risk factor for positional malposition [6]. History of previous intubation difficulty was similarly not captured in our dataset. The absence of these variables limits the completeness of the multivariable model and may have introduced residual confounding. Anesthesiologist experience was categorized by duration of post-residency clinical practice rather than procedural volume. The number of LDLT placements or thoracic anesthesia cases performed was not recorded and could not be retrieved retrospectively. Procedural volume may more precisely reflect technical competence than time-based categorization, and this represents a further constraint on the interpretability of the experience categories. The single-center design did, however, ensure uniform application of the FOB trigger criteria and a standardized clinical environment throughout the study period. A key interpretive constraint deserves emphasis. FOB was performed only when clinical findings indicated possible malposition. The study therefore identifies predictors of clinically indicated bronchoscopy, not predictors of LDLT malposition per se. Patients with subclinical malposition that generated no clinical trigger were not captured, and this group cannot be quantified retrospectively. Prior studies using routine FOB have demonstrated malposition rates of 32–48% despite satisfactory clinical assessment [9, 21, 22], with more than 90% of malpositions going unrecognized by the placing anesthesiologist [4]. The 7.05% clinically triggered FOB rate in our cohort represents the symptomatic fraction of a substantially larger underlying malposition pool.

Nevertheless, the large cohort size, the high-volume real-world setting, and the uniform institutional protocol strengthen the clinical relevance of our findings.

## Conclusion

In this high-volume thoracic surgery cohort managed with a selective bronchoscopy strategy, anesthesiologist experience emerged as the only independent predictor of intraoperative FOB requirement after LDLT placement. Although several patients and procedure related variables were associated with FOB use in univariable analyses, operator experience remained the dominant determinant in multivariable modeling. These findings support an experience-guided, selective approach to bronchoscopy, which may enable more targeted utilization of bronchoscopic resources, particularly in centers where routine FOB confirmation is not universally implemented.

## Data Availability

The datasets used and/or analyzed during the current study available from the corresponding author on reasonable request.

## Abbreviations

%: Percentage
ASA: American Society of Anesthesiologists
AUC: Area under the receiver operating characteristic curve
BMI: Body mass index
CI: Confidence interval
FOB: Fiberoptic bronchoscopy
IQR: Interquartile range
LDLT: Left-sided double-lumen tube
LDP: Lateral decubitus position
n: Number
OLV: One-lung ventilation
OR: Odds ratio
SD: Standard deviation
VATS: Video-assisted thoracic surgery

## References

[1] Fischer GW, Cohen E. An update on anesthesia for thoracoscopic surgery. Curr Opin Anaesthesiol. 2010;23(1):7–11. 19907314 10.1097/ACO.0b013e3283346c6d

[2] Bernasconi F, Piccioni F. One-lung ventilation for thoracic surgery: current perspectives. Tumori. 2017;103(6):495–503. 28604996 10.5301/tj.5000638

[3] Shah SB, Hariharan U, Chawla R. Choosing the correct-sized adult double-lumen tube: Quest for the holy grail. Ann Card Anaesth. 2023;26(2):124–32. 37706375 10.4103/aca.aca_140_22 PMC10284481

[4] Campos JH, Hallam EA, Van Natta T, Kernstine KH. Devices for lung isolation used by anesthesiologists with limited thoracic experience: comparison of double-lumen endotracheal tube, Univent torque control blocker, and Arndt wire-guided endobronchial blocker. Anesthesiology. 2006;104(2):261–6. 16436844 10.1097/00000542-200602000-00010

[5] Bussières JS, Slinger P. Correct positioning of double-lumen tubes. Can J Anaesth. 2012;59(5):431–6. 22395826 10.1007/s12630-012-9689-5

[6] Inoue S, Nishimine N, Kitaguchi K, Furuya H, Taniguchi S. Double lumen tube location predicts tube malposition and hypoxaemia during one lung ventilation. Br J Anaesth. 2004;92(2):195–201. 14722168 10.1093/bja/aeh055

[7] Seo JH, Bae JY, Kim HJ, Hong DM, Jeon Y, Bahk JH. Misplacement of left-sided double-lumen tubes into the right mainstem bronchus: incidence, risk factors and blind repositioning techniques. BMC Anesthesiol. 2015;15:1–7. 26511934 10.1186/s12871-015-0138-1 PMC4625646

[8] Parab SY, Divatia JV, Chogle A. A prospective comparative study to evaluate the utility of lung ultrasonography to improve the accuracy of traditional clinical methods to confirm position of left sided double lumen tube in elective thoracic surgeries. Indian J Anaesth. 2015;59(8):476–81. 26379290 10.4103/0019-5049.162983 PMC4551024

[9] Gupta E, Singh P, Tejpal Karna S, Niwariya Y, Waindeskar V, Jain S, et al. Comparison of lung ultrasound technique versus clinical method to evaluate the accuracy of size and placement of left endobronchial double lumen tube in patients undergoing elective thoracic surgery: a prospective observational study. Monaldi Arch Chest Dis. 2023;94(3):1–7. 10.4081/monaldi.2023.2700 37731374

[10] Doğan A, Sazak H, Tunc M, Alagöz A. Effectiveness Thoracic Ultrasonography to Confirm Position of Double Lumen Endobronchial tube. J Cardio-Vascular-Thoracic Anaesth Intensive Care Soc. 2019;25(4):247–56.

[11] Wang PK, Lin TY, Su IM, Chang KV, Wu WT, Özçakar L. Preoperative lung ultrasound for confirming the double-lumen endotracheal tube position for one-lung ventilation: A systematic review and meta-analysis. Heliyon. 2023;9(4):1–9. 10.1016/j.heliyon.2023.e15458 PMC10147981 37128322

[12] Elsabeeny WY, Ibrahim MA, Abed SM, Shehab NN. Role of Lung Ultrasound in Confirmation of Double Lumen Endotracheal Tube Placement for Thoracic Surgeries: A Prospective Diagnostic Accuracy Study. Anesth Pain Med. 2022;12(5):1–7. 10.5812/aapm-132312 PMC10016132 36937173

[13] Brodsky JB. Fiberoptic bronchoscopy need not be a routine part of double-lumen tube placement. Curr Opin Anaesthesiol. 2004;17(1):7–11. 17021523 10.1097/00001503-200402000-00003

[14] de Bellis M, Accardo R, Di Maio M, La Manna C, Rossi GB, Pace MC, et al. Is flexible bronchoscopy necessary to confirm the position of double-lumen tubes before thoracic surgery? Eur J Cardiothorac Surg. 2011;40(4):912–6. 21802958 10.1016/j.ejcts.2011.01.070

[15] Granell Gil M, Martínez Plumed R. Airway management in thoracic anesthesia in the light of the guidelines of EACTAIC-thoracic group: what is next? Curr Opin Anaesthesiol. 2026;39(1):66–70. 41222996 10.1097/ACO.0000000000001586

[16] Do YW, Kim JH, Kim K, Oh J, Kwak KH, Jeon Y, et al. Effect of Minimum Bronchial Cuff Volume of Left-Sided Double-Lumen Tube for One-Lung Ventilation on the Change in Bronchial Cuff Pressure during Lateral Positioning in Thoracic Surgery: A Prospective Observational Study. J Clin Med. 2023;12(7):1–10. 10.3390/jcm12072473 PMC10095022 37048557

[17] Kim JH, Kim E, Kim IY, Choi EJ, Byun SH. Changes in the Bronchial Cuff Pressure of Left-Sided Double-Lumen Endotracheal Tube by Lateral Positioning: A Prospective Observational Study. J Clin Med. 2021;10(8):1–10. 10.3390/jcm10081590 PMC8070095 33918748

[18] Chen ZY, Lin YM, Wu JH, Fu YY, Xu XT, Li Y, et al. Does the periportal end of a double-lumen endobronchial tube need to be fixed to prevent dislocation of the cuffed end caused by a change in position? A randomized controlled trial. Ann Med. 2023;55(2):1–10. 37619404 10.1080/07853890.2023.2247422 PMC10453979

[19] Eldawlatly AA. Double lumen tube: Size and insertion depth. Saudi J Anaesth. 2021;15(3):280–2. 34764835 10.4103/sja.sja_192_21 PMC8579509

[20] Palaczynski P, Misiolek H, Bialka S, Owczarek AJ, Gola W, Szarpak Ł, et al. A randomized comparison between the VivaSight double-lumen tube and standard double-lumen tube intubation in thoracic surgery patients. J Thorac Dis. 2022;14(10):3903–14. 36389329 10.21037/jtd-22-451 PMC9641341

[21] Onifade A, Lemon-Riggs D, Smith A, Pak T, Pruszynski J, Reznik S, et al. Comparing the rate of fiberoptic bronchoscopy use with a video double lumen tube versus a conventional double lumen tube-a randomized controlled trial. J Thorac Dis. 2020;12(11):6533–41. 33282355 10.21037/jtd-20-1595 PMC7711371

[22] Sazak H, Göktaş U, Alagöz A, Demirbaş ÇS, Güven Ö, Şavkılıoğlu E. Comparison of two different right-sided double-lumen tubes with different designs. Turk J Med Sci. 2012;42(6):1063–9.

[23] Granell M, Petrini G, Kot P, Murcia M, Morales J, Guijarro R, et al. Intubation with vivasight double-lumen tube versus conventional double-lumen tube in adult patients undergoing lung resection: A retrospective analysis. Ann Card Anaesth. 2022;25(3):279–85. 35799554 10.4103/aca.aca_43_21 PMC9387622

[24] Somma J, Couture ÉJ, Pelletier S, Provencher S, Moreault O, Lohser J, et al. Non-ventilated lung deflation during one-lung ventilation with a double-lumen endotracheal tube: a randomized-controlled trial of occluding the non-ventilated endobronchial lumen before pleural opening. Can J Anaesth. 2021;68(6):801–11. 33797018 10.1007/s12630-021-01957-9

[25] Ryu T, Kim E, Kim JH, Woo SJ, Roh WS, Byun SH. Comparing the placement of a left-sided double-lumen tube via fiberoptic bronchoscopy guidance versus conventional intubation using a Macintosh laryngoscope, to reduce the incidence of malpositioning: study protocol for a randomized controlled pilot trial. Trials. 2019;20(1):1–8. 30646931 10.1186/s13063-018-3163-9 PMC6334414

[26] Rispoli M, Calgaro G, Strano G, Rosboch GL, Massullo D, Piccirillo F, et al. Deciding how to decide the correct double-lumen tube: a narrative review of methods and evidence. J Anesth Analg Crit Care. 2025;5(1):1–10. 41088471 10.1186/s44158-025-00286-3 PMC12522624

[27] Rastogi A. Practice patterns of left-sided double-lumen tube: Does It match recommendation from literature. Ann Card Anaesth. 2021;24(1):47–8. 33938831 10.4103/aca.ACA_165_18 PMC8081118

[28] Kinra M, Parab S, Shetmahajan M, Salunke B, Ranganathan P. Practices of Sizing of Left-Sided Double Lumen Tubes at a Tertiary-Referral Cancer Centre in India: A Retrospective Cohort Study. Ann Card Anaesth. 2025;28(4):427–31. 41081685 10.4103/aca.aca_24_25 PMC12591289

[29] Hennessy S, Bilker WB, Berlin JA, Strom BL. Factors influencing the optimal control-to-case ratio in matched case-control studies. Am J Epidemiol. 1999;149(2):195–7. 9921965 10.1093/oxfordjournals.aje.a009786

[30] Klein U, Karzai W, Bloos F, Wohlfarth M, Gottschall R, Fritz H, et al. Role of fiberoptic bronchoscopy in conjunction with the use of double-lumen tubes for thoracic anesthesia: a prospective study. Anesthesiology. 1998;88(2):346–50. 9477054 10.1097/00000542-199802000-00012

[31] Nguyen RD, Kurnutala LN, Tucci MA, Hierlmeier BJ. Comparison of different size left-sided double-lumen tubes for thoracic surgery. Ann Card Anaesth. 2021;24(1):42–6. 33938830 10.4103/aca.ACA_93_19 PMC8081122

[32] Cui G, Zhao L, Chi C, Liang S, Liu Z. The feasibility and accuracy of the method for selecting the optimal size of double-lumen tube in thoracic surgery: a prospective, randomized controlled trial. Sci Rep. 2024;14(1):1–7. 39080380 10.1038/s41598-024-68349-z PMC11289487

[33] Zani G, Stefano M, Tommaso BF, Marco R, Salvatore B, Antonio C, et al. How clinical experience leads anesthetists in the choice of double-lumen tube size. J Clin Anesth. 2016;32:1–3. 27290933 10.1016/j.jclinane.2015.12.030

[34] Piccioni F, Droghetti A, Bertani A, Coccia C, Corcione A, Corsico AG, et al. Recommendations from the Italian intersociety consensus on Perioperative Anesthesia Care in Thoracic surgery (PACTS) part 1: preadmission and preoperative care. Perioper Med (Lond). 2020;9(1):1–17. 33292657 10.1186/s13741-020-00168-y PMC7704118

[35] Campos JH, Hallam EA, Ueda K. Training in placement of the left-sided double-lumen tube among non-thoracic anaesthesiologists: intubation model simulator versus computer-based digital video disc, a randomised controlled trial. Eur J Anaesthesiol. 2011;28(3):169–74. 21088594 10.1097/EJA.0b013e328340c332

[36] Pennefather SH, Russell GN. Placement of double lumen tubes–time to shed light on an old problem. Br J Anaesth. 2000;84(3):308–10. 10793587 10.1093/oxfordjournals.bja.a013430

[37] Levy-Faber D, Malyanker Y, Nir RR, Best LA, Barak M. Comparison of VivaSight double-lumen tube with a conventional double-lumen tube in adult patients undergoing video-assisted thoracoscopic surgery. Anaesthesia. 2015;70(11):1259–63. 26192743 10.1111/anae.13177

